# Inhibition of activin-like kinase 4/5 attenuates cancer cachexia associated muscle wasting

**DOI:** 10.1038/s41598-019-46178-9

**Published:** 2019-07-08

**Authors:** S. Levolger, E. A. C. Wiemer, J. L. A. van Vugt, S. A. Huisman, M. G. van Vledder, S. van Damme-van Engel, G. Ambagtsheer, J. N. M. IJzermans, R. W. F. de Bruin

**Affiliations:** 1 Department of Surgery, Erasmus MC University Medical Centre, Rotterdam, The Netherlands; 2 Medical Oncology, Erasmus MC University Medical Centre, Rotterdam, The Netherlands

**Keywords:** Cancer, Preclinical research

## Abstract

Cancer mediated activation of the ActRIIB-ALK4/5 heterodimer by myostatin is strongly associated with muscle wasting. We investigated *in vitro* and *in vivo* the efficacy of ALK4/5 receptor blockers SB431542 and GW788388 in preventing muscle wasting, and explored synergy with IGF-I analogue LONG R3 (LR3) IGF-I. *In vitro*, C2C12 skeletal muscle cells were treated with vehicle, SB431542, GW788388 and LR3 IGF-I. A C26-CD2F1 cachexia model was used to induce cachexia *in vivo*. Mice were allocated as non-tumour bearing (NTB) or C26 tumour-bearing (C26 TB) vehicle control, treated with SB431542, LR3 IGF-I, SB431542 and LR3 IGF-I, or GW788388 (intraperitoneally or orally). *In vitro*, differentiation index and mean nuclei count increased using SB431542, GW788388, LR3 IGF-I. *In vivo*, GW788388 was superior to SB431542 in limiting loss of bodyweight, grip-strength and gastrocnemius weight. and downregulated Atrogin-1 expression comparable to NTB mice. LR3 IGF-I treatment limited loss of muscle mass, but at the expense of accelerated tumour growth. In conclusion, treatment with GW788388 prevented cancer cachexia, and downregulated associated ubiquitin ligase Atrogin-1.

## Introduction

Progressive skeletal muscle wasting, with or without loss of adipose tissue, is observed in up to 50 per cent of all cancer patients^1,2^. This multifactorial syndrome is known as cachexia, and cannot be fully reversed by conventional nutritional support. Cachexia leads to progressive functional impairment^3^. Up to 20 per cent of all cancer-associated deaths may be attributed to cachexia, through the sequelae of immobility and cardiac or respiratory failure^2,4^. We have shown that skeletal muscle wasting is associated with poor outcome in patients with colorectal and hepatopancreatobiliary malignancies^5–8^. Catabolic cytokines released due to the tumour-host interaction^9^ and miRNA cargo bearing microvesicles^10^ are key pathogenic mechanisms leading to cancer cachexia, further impacted patient factors, including age and levels of physical activity^11,12^.

Myostatin, also known as growth and differentiation factor 8 (GDF-8), is a member of the transforming growth factor beta (TGF-β) superfamily^13^ and is an essential regulator of muscle fibre growth and differentiation, i.e. myostatin limits muscle fibre growth^14–16^. Myostatin has a high affinity for the skeletal muscle cell-surface activin IIB receptor (ActRIIB). After binding to myostatin this receptor forms a heteromeric complex with activin-like kinases four (ALK4) and five (ALK5) and activates the myostatin signal transduction pathway^17–19^, including Smad2/3 and MAPK^20^. Activation of Smad2/3 not only induces an Akt-mediated FoxO-dependent muscle protein breakdown via the ubiquitin-proteasome system but also decreases muscle protein synthesis via inhibition of Akt^21,22^. Disruption of the myostatin gene is associated with gross muscle hypertrophy^23–26^. Likewise, elevated myostatin levels are associated with progressive muscle wasting in chronic obstructive pulmonary disease (COPD), chronic heart failure (CHF), acquired immunodeficiency syndrome (AIDS), liver cirrhosis, ageing, and experimental cancer models^27–32^.

Systemic administration of myostatin induces cachexia in mice^33^, whereas inhibition of myostatin using modified RNA oligonucleotides, systemic administration of the activin receptor extracellular domain/Fc fusion protein (ACVR2B-Fc), and soluble ActRIIB receptor preserve skeletal muscle mass in experimental cancer cachexia^34–37^. Moreover, ALK4 has recently been reported to play a pivotal role in both myogenesis as well as the regulation of protein synthesis and degradation in skeletal muscle cells in mdx muscular dystrophy mice^38^. In contrast to myostatin, insulin-like growth factor 1 (IGF-I) is an important anabolic regulator of muscle fibre growth and differentiation. Via activation of the IGF-I/PI3K/Akt signalling pathway, it not only regulates protein synthesis but also limits upregulation of key mediators of skeletal muscle atrophy, i.e. MuRF1 and FBXO32, more commonly referred to as MAFbx/Atrogin-1^39,40^. Such upregulation of MuRF1 and MAFbx/Atrogin-1 has extensively been reported in experimental cancer models. Recently, it has also been reported to be present in patients with malignancies^41,42^. Furthermore, decreased serum levels of IGF-I have been reported in experimental cancer cachexia and cachectic gastric cancer patients^43,44^. Considering its strong anabolic potential such decrease may aggravate muscle wasting in cancer. Of paramount clinical concern in regard to IGF-I treatment for cancer cachexia is the potential of IGF-I to accelerate tumour growth^45,46^. However, *in vivo* supplementation of similar growth factors, i.e. growth hormone-releasing hormone (GHRH) and a recombinant human IGF-I/insulin-like growth factor binding protein-3 complex (rhIGF-I/IGFBP-3) as a potential treatment for cancer cachexia had no effect on tumour growth^47,48^. In contrast, supplementation of insulin-like growth factors attenuates muscle wasting in experimental cancer cachexia models^48,49^.

Taking these data into consideration, we sought to determine whether (1) systemic inhibition of ALK4/5, and thus potentially blocking the myostatin signalling pathway, enhances myogenesis *in vitro* and limits muscle wasting in experimental cancer cachexia *in vivo*, and (2) whether combined treatment of ALK 4/5 inhibition and IGF-I supplementation would improve treatment outcome without impacting on tumour growth^50^. Our data shows that both ALK4/5 receptor inhibition and LONG R3 IGF-I analogues enhance C2C12 skeletal muscle cell differentiation *in vitro*, successfully limit cancer cachexia *in vivo*, and down-regulates the associated target genes.

## Materials and Methods

### Materials

#### SB431542

SB431542 (Bio-connect BV, Huissen, The Netherlands) is a potent and selective inhibitor of the transforming growth factor-β type I receptors ALK4, ALK5, and ALK7, It has no effect on ERK1, ERK2 or JNK in C2C12 cells in concentrations up to 10 μM^51^. It does however weakly inhibit MAP kinase p38α, but not any of the other p38 MAP kinases^51^. C2C12 cells treated with anti-myogenic TGF- β1 have previously shown full rescue of myogenic effect with the addition of SB431542^52^. And SB431542 has been shown to inhibit myostatin induced C-terminal Smad2 phosphorylation^53^. Dose, schedule and route of administration for *in vivo* experiments are specified in Table 1.

**Table 1:** Dose, schedule, and route of administration for *in vivo* experiments.

	Dose	Schedule	Route of administration (volume)
DMSO (control)	—	Daily	IP (1 μL/g BW)
SB431542	10 mg/kg	Daily	IP (1 μL/g BW)
LONG R3 IGF-I	200 μg	Every other day	IM (50 μL)
GW788388	10 mg/kg	Daily	IP (1 μL/g BW)
GW788388	10 mg/kg	Daily	PO (250 μL)

IP Intraperitoneally. IM Intramuscularly. PO Orally.

#### GW788388

GW788388 (Sigma-Aldrich, St. Louis, The United States of America) is a potent and selective inhibitor of the transforming growth factor-β type I receptor ALK5. GW788388 has a dose-dependent inhibition of TGF- β induced Smad activation and it has no effect on ERK1, ERK2 or p38 MAPK^54^. GW788388 has been shown to be orally active^55^. Dose, schedule and route of administration for *in vivo* experiments are specified in Table 1.

#### LONG R3 IGF-I

LONG R3 IGF-I (Bio-connect BV, Huissen, The Netherlands) is a recombinant analogue of human insulin-like growth factor-I (IGF-I). Dose, schedule and route of administration for *in vivo* experiments are specified in Table 1.

### Cell cultures

Colon-26 (C26) adenocarcinoma cells (kindly provided by Dr. D.O. McCarthy, Ohio State University, Columbus, OH, USA) were maintained in RPMI 1640 (Westburg BV, Leusden, The Netherlands) supplemented with 10% foetal bovine serum (FBS, Sigma-Aldrich, St. Louis, The United States of America), and 1% penicillin/streptomycin (P/S, Fisher Scientific, Waltham, The United States of America) at 37 °C in a 5% carbon dioxide environment. C2C12 muscle myoblast cells were obtained from American Type Culture Collection (ATCC-CRL-1772, ATCC, Manassas, VA, USA) and maintained in growth medium (GM) consisting of DMEM (glutamine)(Westburg BV, Leusden, The Netherlands) supplemented with 10% FBS, and 1% P/S at 37 °C in a 10% carbon dioxide environment. To induce myogenic differentiation, at near-confluence, GM was substituted with differentiation medium (DM) consisting of DMEM supplemented with 2% horse serum (HS) (Fisher Scientific, Waltham, The United States of America), and 1% P/S. DM was routinely changed every 24 h.

### *In Vitro* model

C2C12 cells were plated on 0.1% gelatine-coated coverslips in 6-well plates (3 × 10^4^ cells/cm^2^) and supplemented with GM. Following overnight attachment, GM was substituted with DM with treatment or vehicle (DMSO 0.1%) (Sigma-Aldrich, St. Louis, The United States of America) for up to 6 days (2, 4, and 6 d). Treatment consisted of SB431542 (dosages: 0.1 μM, 1.0 μM, 2.0 μM, or 5.0 μM), GW788388 (dosages: 1.0 μM, 2.0 μM, 5.0 μM, or 10.0 μM), or LR3-IGF-I (5 ng/mL, 10 ng/mL, 20 ng/mL, or 30 ng/mL). Each treatment was performed in triplicate.

### Fusion index

The coverslips were stained with haematoxylin and eosin (H&E) according to standard laboratory protocols. Images were acquired from four predefined fields per well at a magnification of 200x, Differentiation into myotubes was determined by determining the fusion index by manually counting the number of nuclei in multinucleated myotubes, i.e. myotubes with 2 or more nuclei, divided by the total number of nuclei^56^.

### Animal ethics committee approval

All animal experiments were performed with the approval of the Animal Experiments Committee of the Erasmus University Medical Centre, Rotterdam, the Netherlands and in accordance to the Dutch National Experiments on Animal Act, and complied with the EU adopted Directive 86/609/EEC (1986).

### Animals

Male CD2F1 (BALB/c × DBA/2 F1) mice of 8 weeks (~25 grams) were obtained from Charles River, Maastricht, the Netherlands. Upon arrival, animals have been housed in individually ventilated cages and maintained at 22 °C under a 12 h light-dark cycle with ad libitum access to CRM (P) chow (Special Diet Services, Witham, Essex, UK) and water (n = 3–4 animals per cage). Animals were acclimatized for one week prior to the start of the experiments.

### C26 tumour-bearing mice

Animals allocated in tumour bearing (TB) groups received a subcutaneous (SC) inoculation in the right flank with 0.5 × 10^6^ C26 adenocarcinoma cells in 100 μL sterile PBS under anaesthesia by isoflurane inhalation (5% isoflurane induction), a classic model of cancer cachexia^50^.

### Assessment of grip-strength

The effect of treatment on muscular strength was quantified via the widely used grip-strength test of Meyer *et al*.^57^ Combined hind- and forelimb grip strength was measured twice per week by placing the animal on a grid (8 × 8 cm) attached to a force gauge (BIOSEB, Chaville, France). The mice were allowed to grasp on to the grid. Thereafter, the mice were gently pulled by the tail along the sensor axle until grip is released. Grip strength assessment was performed at the same time per day and prior to administration of the investigated treatment agents. Maximum strength produced before releasing the grid was registered in triplicate with one minute rest period for each animal. Obtained values were averaged to provide a mean force measurement for each individual animal and subsequently normalized to each animal’s grip-strength respectively on day 0. All measurements were performed blind with respect to treatment.

### Bodyweight, muscle mass, and tumour size

Bodyweight was recorded daily. Bodyweight was normalized to each animal’s body weight on day 0. Tumour size was recorded every other day starting on day 9 after tumour inoculation using digital callipers. Tumour mass was estimated via the formula mass (mg) = tumour volume (mm^3^) = width^2^ × length/2^58^. Animals were sacrificed by cervical dislocation under isoflurane anaesthesia on day 21 or upon body weight loss exceeding 20%. Gastrocnemius (GCM), tibialis anterior (TA), and soleus (Sol) muscles of both hind legs and tumour were dissected, weighed and immediately snap-frozen in liquid nitrogen and stored at −80 °C until analysis.

### RNA isolation and real-time polymerase chain reaction

Cancer-cachexia associated muscle wasting is known to be most pronounced in fast-twitch type II-containing muscles, such as GCM and TA^59^. Therefore, for gene expression analysis, total RNA was isolated from snap-frozen GCM muscle tissue using Trizol reagent (Invitrogen, Breda, the Netherlands), and subsequently purified by DNase treatment (RQ1 RNase-Free DNase) (Promega Benelux B.V., Leiden, the Netherlands). 1 μg of total RNA was reversed transcribed to cDNA using random hexamer primers (Invitrogen, Breda, the Netherlands), and Superscript II RT (Invitrogen, Breda, the Netherlands). Quantitative real-time polymerase chain reaction (RT-PCR) was performed using an iCycler real-time PCR system (Biorad, California, The United States of America) using SYBR Green (Sigma-Aldrich, St. Louis, The United States of America). Used primer sequences can be found in Table 2. GAPDH, HPRT and HMBS were selected as housekeeping genes for normalization from commonly used housekeeping genes, i.e. ACTB, B2M, GAPDH, HMBS, HPRT, RPL13A, SDHA, TBP, UBC and YHWAZ, after being tested using a gene-stability measure developed by Vandesompele *et al*.^60^ as previously described^61^. The geometric mean was used to average the control genes.

**Table 2:** Reverse transcription-polymerase chain reaction primer sequences.

Gene	Forward Primer	Reverse Primer	Genbank Accession Number
Atrogin1	5′-GTTTTCAGCAGGCCAAGAAG	5′-TTGCCAGAGAACACGCTATG	AF_441120
MyoD	5′-AAACCCCAATGCGATTTATCAGG	5′-TAAGCTTCATCTTTTGGGCGTGA	NM_010866
Myogenin	5′-CACTCCCTTACGTCCATCGT	5′-CAGGACAGCCCCACTTAAAA	NM_031189
Murf1	5′-AGGTGTCAGCGAAAAGCAGT	5′-CCTCCTTTGTCCTCTTGCTG	NM_009066
GAPDH	5′-ATGCATCCTGCACCACCAACT	5′-GCAGTGATGGCATGGACTGTG	NM_008084
Hmbs/Pgbd	5′-GTGCCATTGTCCTGGCTGTG	5′-TGCATTCCTCTGGGTGCAAA	—
HPRT	5′-AAGCAGTACAGCCCCAAAATGG	5′-CCAACAAAGTCTGGCCTGTATCC	—

### Statistics

Categorical data are expressed as number (percentage) and continuous variables as mean ± SEM (normal distribution, visually assessed and by means of the Shapiro-Wilks test) or median and (range). Body weight and grip-strength were normalized to each animal’s body weight and grip-strength respectively on day 0. Muscle weight from the left hind leg and right hind leg were averaged to provide a mean muscle weight (GCM, TA, and Sol) for each animal. We tested the difference between healthy animals and untreated, TB animals using an unpaired t-test. Multiple group comparisons were done by one-way ANOVA with a Bonferroni’s post hoc test. Spearman-Rho rank correlation coefficient was used for testing bivariate correlations. All analyses were performed using IBM SPSS Statistics for Windows, version 21.0 (IBM Corp., Armonk, NY, USA). A *P* value < 0.05 was considered statistically significant.

The datasets generated during and/or analysed during the current study are available from the corresponding author on reasonable request.

## Results

### SB431542, GW788388, and LONG R3 IGF-I enhance myogenesis in C2C12 cells

**Figure 1:**
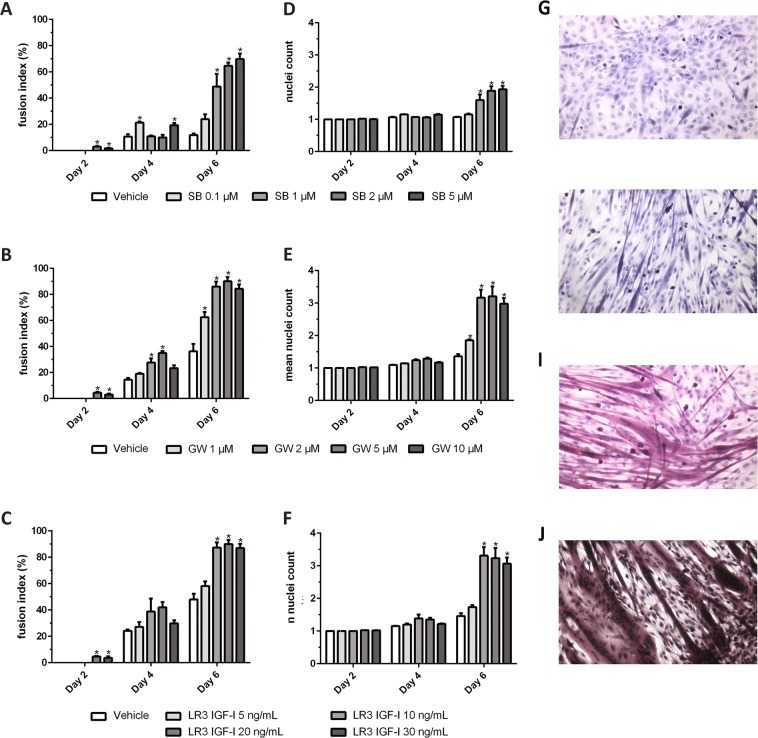
Fusion indices of C2C12 cells treated with SB431542, GW788388 and LONG R3 IGF-I. Fusion indices of C2C12 cells on days two, four, and six for (**A**) different concentrations of SB431542 (SB), (**B**) GW788388 (GW), and (**C**) LONG R3 IGF-I (LR3 IGF-I) and mean nuclei count for (**D**) SB431542, (**E**) GW788388, and (**F**) LONG R3 IGF-I. *A statistically significant difference (p < 0.05 of two-way ANOVA followed by a post hoc Bonferroni test) was observed compared to vehicle samples on the corresponding day. Representative images of H&E stained differentiated (**G**) vehicle treated C2C12 cells, (**H**) SB431542 treated cells, (**I**) GW788388 treated cells, and (**J**) LONG R3 IGF-I treated C2C12 cells. All acquired images were taken from day 6 samples.

We investigated the efficacy of ALK4/5 inhibitors SB431542 and GW788388, and the IGF-I analogue LONG R3 IGF-I, *in vitro* using the C2C12 skeletal muscle cell model. C2C12 myoblasts were cultured in differentiation medium (DM) supplemented with ALK4/5 inhibitors SB431542 (Fig. 1A), or GW788388 (Fig. 1B) during six days. In groups treated with SB431542 1 µM (48.8% ± 19.6, p = 0.001), 2 µM (64.6% ± 5.2, p < 0.001), or 5 µM (69.8% ± 8.4, p < 0.001) differentiation into myotubes as determined by the fusion index on day 6 was significantly higher compared to vehicle treated controls (11.8% ± 2.4). There were no statistically significant differences between these three treatment concentrations. However, fusion index on day 6 was significantly higher in groups treated with SB431542 1 µM (p = 0.042), 2 µM (p = 0.001), or 5 µM (p < 0.001) compared to treatment with 0.1 µM (24.0% ± 7.1). The mean number of nuclei in these cells were 1.1 in the vehicle group, compared to groups treated with SB431542 0.1 µM, 1.2 (p > 0.999), 1 µM, 1.6 (p < 0.001), 2 µM, 1.9 (p < 0.001), and 5 µM, 1.9 (p < 0.001) (Fig. 1D).

GW788388 enhanced differentiation of C2C12 comparable to, if not better than, SB431542 (Fig. 1A,B). In groups treated with 1 µM (62.5% ± 7.8, p = 0.004), 2 µM (86.1% ± 7.4, p < 0.001), 5 µM (90.1% ± 6.6, p < 0.001), and 10 µM GW788388 (84.3% ± 6.7, p < 0.001) the fusion index on day 6 was significantly higher compared to vehicle treated control cells (36.3% ± 11.4). There were no statistically significant differences between treatment with 2 µM, 5 µM, and 10 µM. However, fusion index on day 6 was significantly higher in groups treated with GW788388 2 µM (p = 0.010), 5 µM (p = 0.002), or 10 µM (p = 0.019) compared to treatment with 1 µM. The mean number of nuclei in these cells was 1.4 in the vehicle group, compared to groups treated with GW788388 1 µM, 1.9 (p = 0.014), 2 µM, 3.2 (p < 0.001), 5 µM, 3.2 (p < 0.001), and 10 µM, 3.0 (p < 0.001) (Fig. 1E).

LONG R3 IGF-I gave similar results as GW788388 treatment (Fig. 1C). In groups treated with LONG R3 IGF-I 10 ng/mL (87.3% ± 7.9, p < 0.001), 20 ng/mL (90.0% ± 6.5, p < 0.001), or 30 ng/mL (87.0% ± 6.4, p < 0.001) the fusion index was significantly higher compared to vehicle treated control cells (48.0% ± 8.9). There were no statistically significant differences between these three treatment concentrations. However, fusion index on day 6 was significantly higher in groups treated with LONG R3 IGF-I 10 ng/mL (p = 0.001), 20 ng/mL (p < 0.001), or 30 ng/mL (p = 0.001) compared to 5 ng/mL (58.2% ± 6.9). The mean number of nuclei in these cells was 1.5 in the vehicle group, compared to groups treated with LONG R3 IGF-I 5 ng/mL, 1.6 (p = 0.513), 10 ng/mL, 3.3 (p < 0.001), 20 ng/mL, 3.2 (p < 0.001), and 30 ng/mL, 3.1 (p < 0.001) (Fig. 1F).

The observed differences in fusion index and increase in mean nuclei as a surrogate measurement for muscle hypertrophy suggest enhanced myogenesis in favour of GW788388 and LONG R3 IGF-I compared with SB431542. Representative light microscopy images of the vehicle (0.1% DMSO) treated-, SB431542 treated-, LONG R3 IGF-I treated-, and GW788388 treated C2C12 cells can be found in Fig. 1G–J.

### Treatment with ALK4/5 inhibitors limits muscle wasting and loss of grip-strength in C26 TB mice

**Figure 2:**
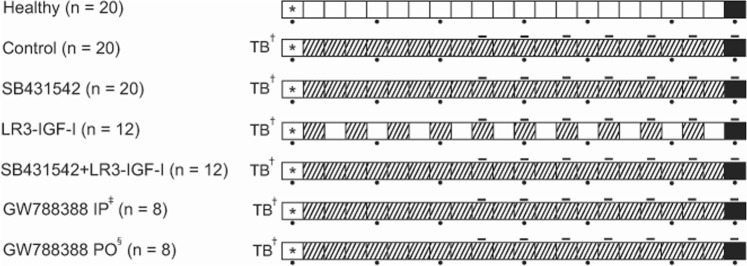
Experimental groups and timeline. Allocation of male CD2F1 mice to different treatment groups. Each box represents a day, ranging from day 0 to day 21. *Start of the experiment (day 0). ^†^Inoculation on day 0 of C26 cells in Tumour-Bearing animals. ^‡^Intraperitoneal. ^§^Orally. Striped boxes indicate day of treatment. Black dots below boxes indicate time points of grip-strength measurement. Lines above boxes indicate time points of tumour size measurement. Black boxes indicate the end of the experiment.

**Figure 3:**
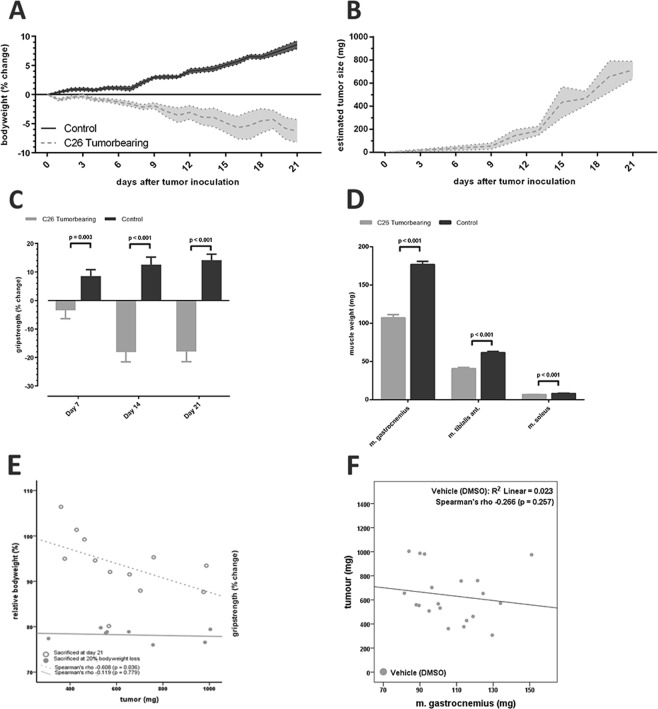
*In vivo* C26 - CD2F1 cachexia model (impact on body weight, tumour size, grip-strength and muscle mass). Data depicted in red indicates C26 tumour-bearing, vehicle treated male CD2F1 mice (n = 20). Data depicted in green indicates healthy, non-tumour-bearing male CD2F1 mice (n = 20). (**A**) Loss of bodyweight (mean ± SEM) in vehicle-treated, C26 tumour-bearing (TB) mice (p < 0.001). (**B**) Estimated tumour growth rate as measured every other day, starting on day 9, via digital callipers. (**C**) Relation between tumour mass and relative bodyweight change. No relation could be observed for animals experiencing rapid bodyweight loss requiring early sacrifice (n = 8, Spearman’s rho = −0.119, p = 0.779). In animals not (yet) experiencing this rapid decline of bodyweight loss prior to day 21, tumour mass was negatively associated with change in bodyweight (n = 12, Spearman’s rho = −0.608, p = 0.036). (**D**) Untreated C26 TB male CD2F1 mice (n = 20) experienced loss of grip-strength throughout the experiments as compared with non-tumour-bearing male CD2F1 mice (n = 20). Student’s t-test was conducted performed for on the mean differences of grip-strength change. This loss of grip-strength could already be observed on day 7 (p = 0.003), but became more apparent on day 14 (p < 0.001). (**E**) Student’s t-test was conducted performed for on the mean weight of m. gastrocnemius, m. tibialis anterior and m. soleus. Wet muscle weight of gastrocnemius (p < 0.001), tibialis anterior (p < 0.001), and soleus (p < 0.001) muscles was significantly reduced in untreated, C26 TB male CD2F1 mice (n = 20) as compared with non-tumour-bearing male CD2F1 mice (n = 20).

Following the favourable *in vitro* results of ALK 4/5 inhibition and LONG R3 IGF-I treatment, we sought to investigate whether treatment with either of these substances alone, or a combination thereof may limit muscle wasting in cancer cachexia. For this purpose, one hundred male CD2F1 mice were allocated to seven groups (Fig. 2). Mice allocated in tumour-bearing groups were inoculated subcutaneously with 0.5 × 10^6^ C26 adenocarcinoma cells. The tumour-bearing mice receiving vehicle treatment experienced hallmark features of cancer cachexia, including progressive body weight loss, loss of grip strength and muscle weight loss (Fig. 3). One day after tumour inoculation allocated mice received a daily intraperitoneal injection with ALK 4/5 inhibitors SB431542 (SB, 10 mg/kg) or GW788388 (GW IP, 10 mg/kg). Considering GW788388 is orally active^55^, an additional group of mice received GW788388 via oral gavage (GW PO, 10 mg/kg).

**Figure 4:**
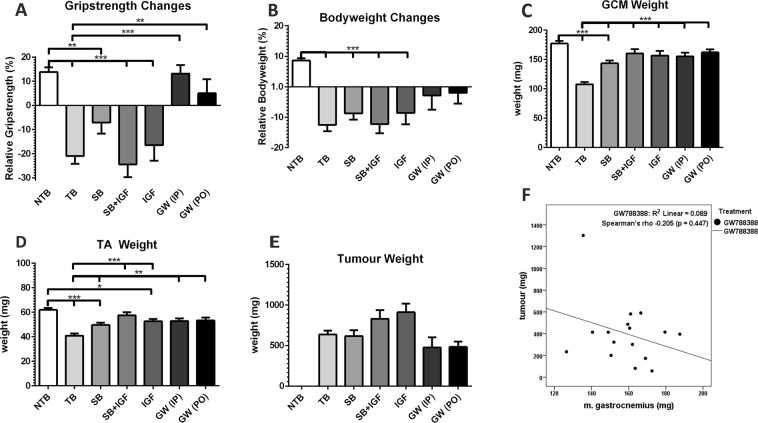
Treatment efficacy of SB431542, GW788388 and LONG R3 IGF-I on bodyweight, grip strength, tumour weight and muscle weight at sacrifice. Bar graphs depicting the mean ± SEM (**A**) relative grip strength immediately prior to sacrifice, (**B**) relative bodyweight at sacrifice, (**C**) gastrocnemius (GCM) muscle weight, (**D**) tibialis anterior (TA) muscle weight, (**E**) tumour weight for non-tumour bearing (NTB) (n = 20); C26 tumour-bearing (TB) vehicle treated (n = 20); SB431542 treated (SB, n = 20); SB431542 *with* LONG R3 IGF-I (SB + IGF, n = 12); LONG R3 IGF-I treated (IGF, n = 12) and GW788388 (GW) treated intraperitoneally (IP, n = 8) and orally (PO, n = 8) male CD2F1 mice. Multiple group comparisons were done by one-way ANOVA with a Bonferroni’s post-hoc test. All groups were compared against NTB mice and TB vehicle treated mice. Asterisk brackets are displayed for significant results only. *p < 0.05 **p < 0.01 ***p < 0.001.

**Figure 5:**
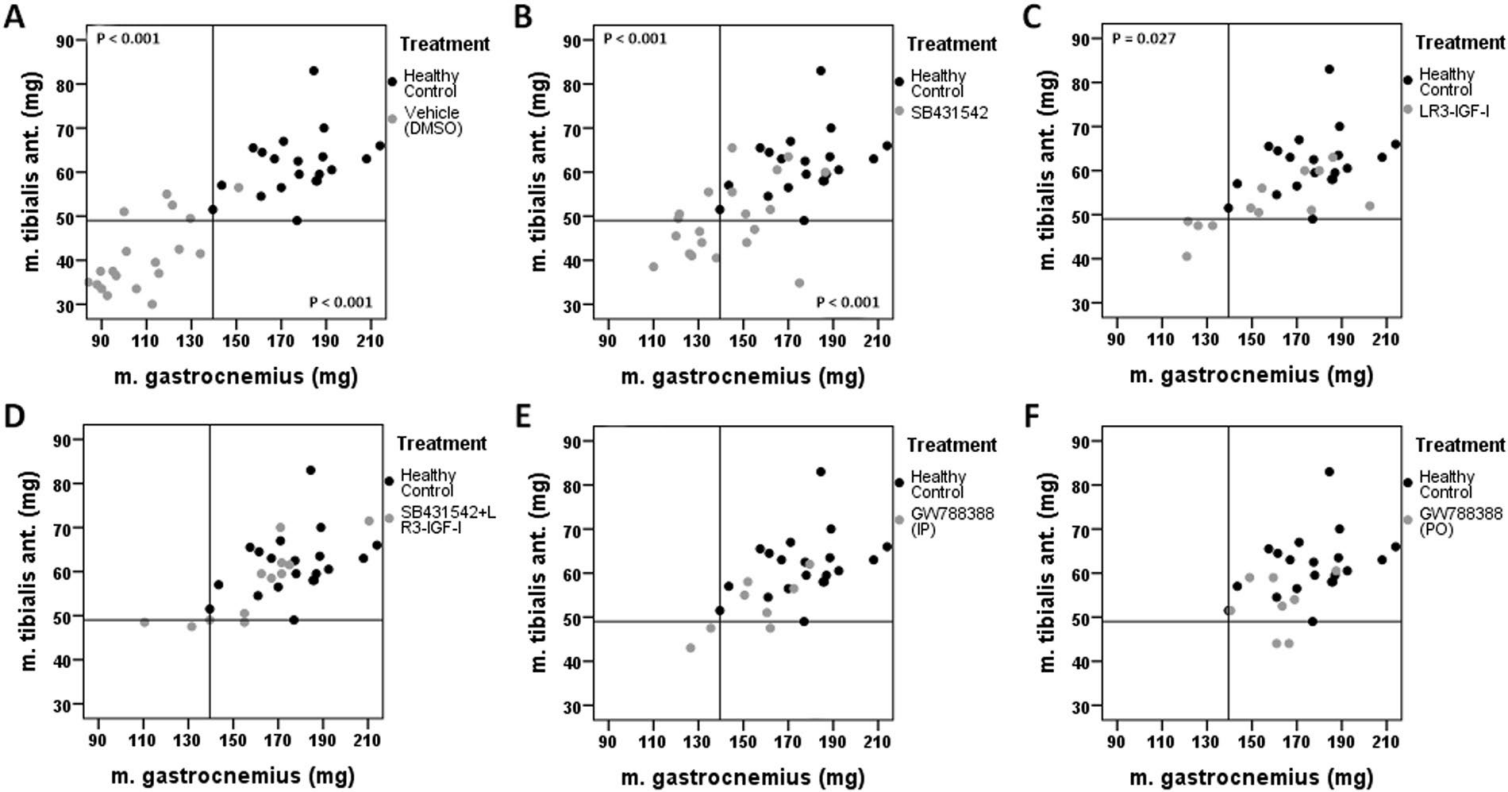
Scatterplot charts for individual m. gastrocnemius and m. tibialis ant. muscle weight in healthy, non-tumour bearing mice and treated tumour-bearing mice. Scatterplots of gastrocnemius (GCM) muscle tibialis anterior (TA) muscle weight for all individual tumour-bearing (TB) groups compared with the non-tumour bearing (NTB) male CD2F1 mice. The horizontal reference line indicates the lowest TA muscle weight observed in NTB mice. The vertical reference line indicates the lowest GCM muscle weight observed in NTB mice. Multiple group comparisons were done by one-way ANOVA with a Bonferroni’s post-hoc. All groups were compared against NTB male CD2F1 mice (n = 20). Differences were observed for (**A**) untreated, C26 TB (n = 20, reduced TA, p < 0.001; reduced GCM, p < 0.001), (**B**) SB431542 treated (n = 20, reduced TA, p < 0.001; reduced GCM, p < 0.001) and (**C**) LONG R3 IGF-I treated male CD2F1 mice (n = 12, reduced TA, p = 0.027). No differences in muscle weight were observed for (**D**) combined SB431542 and LONG R3 IGF-I treated (n = 12), (**E**) GW788388 (intraperitoneally, n = 8) and (**F**) GW788388 (orally, n = 8) treated male CD2F1 mice.

Throughout the experiment, grip-strength of GW PO mice, GW IP mice, and NTB mice was comparable (p > 0.05 at all timepoints, Fig. 4A). SB treated mice and untreated C26 TB mice experienced a statistically significant loss of grip-strength starting on day 14 compared to NTB mice (p = 0.006 and p < 0.001 respectively) which remained present until sacrifice (Fig. 4A). Loss of bodyweight was present in SB mice (−8.6% ± 9.2, p < 0.001) and untreated, C26 TB mice (−12.5% ± 9.4, p < 0.001), but not in GW PO and GW IP treated mice (Fig. 4B). These differences in efficacy between GW788388 and SB431542 could also be observed in the preservation of muscle mass. GCM muscle mass was significantly higher in mice treated with SB (143.3 ± 20.9 mg, p < 0.001), GW IP (154.9 ± 17.8 mg, p < 0.001), or GW PO (162.1 ± 13.9 mg, p < 0.001) compared to untreated, C26 TB mice (107.3 ± 18.7 mg) (Figs 4C and 5E,F). The results from the TA data analyses were comparable, e.g. no differences were observed in mice treated with GW788388 when compared with healthy mice (Figs 4D and 5E,F). No difference in tumour mass was observed between the treatment groups (Fig. 4E). Collectively, these data show that GW788388 treatment preserves body mass, muscle mass and muscle strength in tumour-bearing cachexia prone mice.

### LONG R3 IGF-I treatment preserves muscle mass in C26 TB mice but may accelerate tumour growth

To study the possible synergy between ALK4/5 inhibition and stimulation of muscle growth and differentiation using an IGF-I analogue, mice received the IGF-I analogue LONG R3 IGF-I (200 μg) administered every other day via intramuscular injection, with or without SB431542 (10 mg/kg, i.p.). Compared to NTB mice, LONG R3 IGF-I treated mice experienced a statistically significant loss of grip-strength starting on day 18 (p = 0.026, data not shown), which remained present until sacrifice (Fig. 4A), and loss of body weight (−8.6% ± 13.0, p < 0.001) (Fig. 4B). LONG R3 IGF-I treated mice had comparable GCM muscle weight (156.4 ± 27.4 mg, p = 0.174) (Figs 4C and 5C), but decreased TA muscle weight (52.3 ± 6.4 mg, p = 0.027) (Figs 4D and 5C). However, LONG R3 IGF-I treatment was superior over TB vehicle-treated animals for both GCM(p < 0.001) and TA muscle weight (p = 0.001) (Fig. 4C,D). Combined LONG R3 IGF-I and SB431542 treatment preserved GCM (160.0 ± 25.1 mg, p = 0.612) and TA muscle weight (57.2 ± 8.4 mg, p = 1.000) compared to NTB mice (Figs 4C,D and 5D). Despite preserving muscle mass, a statistically significant loss of grip-strength was observed starting on day 14 (p = 0.004). This loss of grip-strength remained present until sacrifice (Fig. 4A). Similar to treatment with either LONG R3 IGF-I or SB431542, mice receiving LONG R3 IGF-I and SB431542 experienced loss of body weight (−12.2% ± 10.6, p < 0.001) (Fig. 4B). Although the muscle weight data suggest synergism, the observed loss of grip-strength is unfavourable. Moreover, there was a substantial but non-significant increase in tumour growth in mice receiving LONG R3 IGF-I (911 ± 360 mg, p = 0.08) or LONG R3 IGF-I *with* SB431542 (829 ± 371 mg, p = 0.44) compared to untreated, C26 TB mice (635 ± 218 mg) (Fig. 4E). Therefore, possible synergism between LONG R3 IGF-I and GW788388 was not investigated.

### Treatment with ALK4/5 inhibitors modulates target gene expression

**Figure 6:**
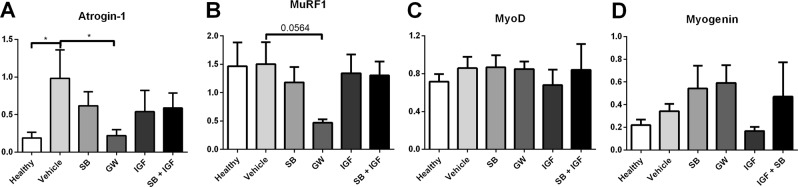
mRNA expression levels in cachectic muscle. Bar graphs depicting the mean ± SEM mRNA expression levels in gastrocnemius muscle of (**A**) Atrogin-1, (**B**) MuRF1, (**C**) MyoD and (**D**) Myogenin in non-tumour bearing (NTB, n = 20); C26 tumour-bearing (TB) vehicle treated (n = 20); tumour-bearing SB431542 treated (SB, n = 20); tumour-bearing combined SB431542 and LONG R3 IGF-I treated (SB + IGF, n = 12); tumour-bearing LONG R3 IGF-I treated (IGF, n = 12) and tumour-bearing GW788388 treated (pooled orally and intraperitoneally GW treated groups, n = 16) male CD2F1 mice. Multiple group comparisons were done by one-way ANOVA with a Bonferroni’s post-hoc test. All groups were compared against NTB mice and TB vehicle treated mice. Asterisk brackets are displayed for significant results only. *p < 0.05.

We determined the mRNA expression levels of E3 ubiquitin ligases, MuRF1 and Atrogin-1, and the two myogenic regulatory factors, MyoD and myogenin, in gastrocnemius muscle samples obtained from the mice at sacrifice (Fig. 6). Atrogin-1 expression was significantly elevated in vehicle-treated animals, but similar to healthy controls in GW788388 treated animals (Fig. 6A). In contrast to Atrogin-1 expression, MuRF1 expression (Fig. 6B) did not increase in tumour-bearing vehicle-treated animals. MuRF1 expression decreased in GW788388 treated animals when compared to vehicle treated animals, although did not quite reach significance (p = 0.056). MyoD (Fig. 6C) and Myogenin (Fig. 6D) expression levels were unaltered by both GW788388 and SB431542 treatment.

## Discussion

Although cachexia is a common finding in patients suffering from a wide variety of malignancies, with detrimental effects on survival as well as the quality of life^8,62^, there currently are no treatment modalities available to successfully halt or reverse its progressive muscle wasting. However, an increasing understanding of the underlying pathways associated with cachexia is shaping the building blocks between science and clinical practice. The suggested role of myostatin in the development of cachexia^33^, as well as the heteromeric receptor complex it binds to^17–19^, have allowed for the first preclinical studies aimed to develop and identify treatment modalities to counter progressive muscle loss. Our study is the first to study the role of two ALK 4/5 inhibitors, in the treatment of cancer-associated muscle loss. The present findings show that pharmacological inhibition of ALK 4/5 successfully limits the occurrence of cancer-associated sarcopenia. And although an associated between tumour mass and body weight was observed, no associated between tumour mass and muscle weight could be found. As such, potential differences in muscle weight cannot be explained by observed non-significant differences in tumour mass.

We hypothesized this inhibition to work based on prior research which detailed the myostatin signalling pathway, wherein myostatin has a high affinity for the ActRIIB receptor, after binding this receptor forms a heteromeric complex with ALK4 and ALK5, subsequently activating the myostatin signal transduction pathway^17–19^, including Smad2/3 and MAPK^20^, which in turn induces an Akt-mediated FoxO-dependent muscle protein breakdown via the ubiquitin-proteasome system^21,22^. In the current study in which we blocked the ALK4 and ALK5 receptors, thus prohibiting the formation of the heteromeric complex, we observed a differential expression of the target genes (i.e. E3 ubiquitin ligases MuRF1 and Atrogin-1) as expected. These findings of preserved muscle mass by targeting the myostatin signalling pathway are in line with previous studies that found muscle mass preservation by using modified RNA oligonucleotides targeting myostatin mRNA, systemic administration of the activin receptor extracellular domain/Fc fusion protein (ACVR2B-Fc), and a soluble ActRIIB receptor^34–37^. Interestingly, the myostatin pathway might not be the only pathway involved in cancer cachexia. Recent studies found the JAK2/STAT3 pathway to be another candidate for pharmaceutical agents to limit muscle wasting in experimental cancer cachexia^63–65^.

In contrast to the successful reduction of muscle wasting by ALK4/5 inhibition, a beneficial synergistic role of the IGF-I analogue LONG R3 IGF-I was not found. Despite a positive effect on wet muscle weight, no effect on muscle strength was found. Despite comparable muscle weight to GW treated mice, LONG R3 IGF-I treated mice had a reduction in body weight. The exact nature of this difference is not known, as no body-composition analysis was performed in this study. Since a trend towards increased tumour growth was observed this was not further investigated. Although this trend was not observed in earlier studies using similar agents^47,48^, we consider this finding to be of importance. The possibility of enhanced tumour growth through IGF-I treatment precludes its use in the clinic. The role of the IGF system in cancer has been investigated in depth for approximately half a century, from which the association between IGF-I and oncogenesis became apparent. Certain malignancies are found to be more prone to being driven by IGF-I, e.g. prostate cancer, colon cancer and lung cancer^66^. Novel treatment strategies are being developed targeting this IGF system, although with mixed results^67^. Systemic treatment with pharmaceutical agents aimed at inhibiting the IGF system may risk worsening cachexia, by concurrent targeting of the anabolic muscle pathways. Although the impact of inhibiting IGF signalling on cachexia has not purposefully been investigated, muscle weakness is reported as a side-effect for such treatment^68^. Future development of muscle-specific anabolic agents without a tumour promoting effect might overcome this drawback.

Several limitations apply to the present study. The study was powered on an expected reduction in loss of muscle weight. As such, non-significant differences in secondary outcome parameters (e.g. total body weight, muscle strength and relative mRNA expression levels) may have been subject to type II errors. Furthermore, survival was not included as one of the endpoints due to the strict ethical guidelines associated with the initiation of this study. Although Zhou *et al*. have previously reported a direct relationship between muscle wasting and survival in a comparable cancer cachexia model^35^, it is therefore, unknown whether the preservation of muscle mass is associated with increased survival in our study. Moreover, the available inhibitors of ALK 4 and ALK 5 as used in this study are preclinical drugs and cannot be directly validated in humans.

In conclusion, this study found that inhibition of ALK 4 and ALK 5 limited muscle wasting in a mouse model of cancer-associated cachexia and reduced the expression of cachexia associated ubiquitin ligase Atrogin-1. The results obtained in the current study are promising and contribute to a growing body of evidence which suggests that muscle wasting in cancer cachexia might be limited by blocking the myostatin signalling pathway. This knowledge may benefit in the selection and development of drug candidates for clinical trials for the treatment of cancer cachexia.
